# Current approaches of the management of mercury poisoning: need of the hour

**DOI:** 10.1186/2008-2231-22-46

**Published:** 2014-06-02

**Authors:** Mehrdad Rafati-Rahimzadeh, Mehravar Rafati-Rahimzadeh, Sohrab Kazemi, Ali Akbar Moghadamnia

**Affiliations:** 1 Department of Nursing, Babol University of Medical Sciences, Babol, Iran; 2 Department of Medical Physics, Kashan University of Medical Sciences, Kashan, Iran; 3 Department of Pharmacology, Faculty of Medicine, Babol University of Medical Sciences, Babol, Iran; 4 Cellular and Molecular Research Center, Babol University of Medical Sciences, Babol, Iran

**Keywords:** Mercury compounds, Chelating agents, Poisoning, Gold nanoparticles, Natural biologic scavengers

## Abstract

Mercury poisoning cases have been reported in many parts of the world, resulting in many deaths every year. Mercury compounds are classified in different chemical types such as elemental, inorganic and organic forms. Long term exposure to mercury compounds from different sources e.g. water, food, soil and air lead to toxic effects on cardiovascular, pulmonary, urinary, gastrointestinal, neurological systems and skin. Mercury level can be measured in plasma, urine, feces and hair samples. Urinary concentration is a good indicator of poisoning of elemental and inorganic mercury, but organic mercury (e.g. methyl mercury) can be detected easily in feces. Gold nanoparticles (AuNPs) are a rapid, cheap and sensitive method for detection of thymine bound mercuric ions. Silver nanoparticles are used as a sensitive detector of low concentration Hg^2+^ ions in homogeneous aqueous solutions. Besides supportive therapy, British anti lewisite, dimercaprol (BAL), 2,3-dimercaptosuccinic acid (DMSA. succimer) and dimercaptopropanesulfoxid acid (DMPS) are currently used as chelating agents in mercury poisoning. Natural biologic scavengers such as algae, azolla and other aquatic plants possess the ability to uptake mercury traces from the environment.

## Introduction

Mercury (Hg) atomic number 80, is a liquid metal at room temperature and pressure. Mercury freezes at -38.9°C and boils at 357°C. It is sometimes called quick silver and is easily alloyed with many other metals, such as gold, silver and tin [1]. It exists in the environment in three forms: elemental mercury (poisonous as vapor), organic mercury (methyl mercury and ethyl mercury) and inorganic mercury (mercuric mercury) and all these forms have toxic health effects [2].

In recent years, due to abundant availability of various chemicals, the rate of intoxication has been surprisingly increased [3,4]. People can overuse or misuse drugs, chemicals, and may get poisoned intentionally or accidentally [5,6]. Similarly, heavy metals, either released from natural sources or from industries wastes pose a consistent health threat to human being [7].

**Figure 1:**
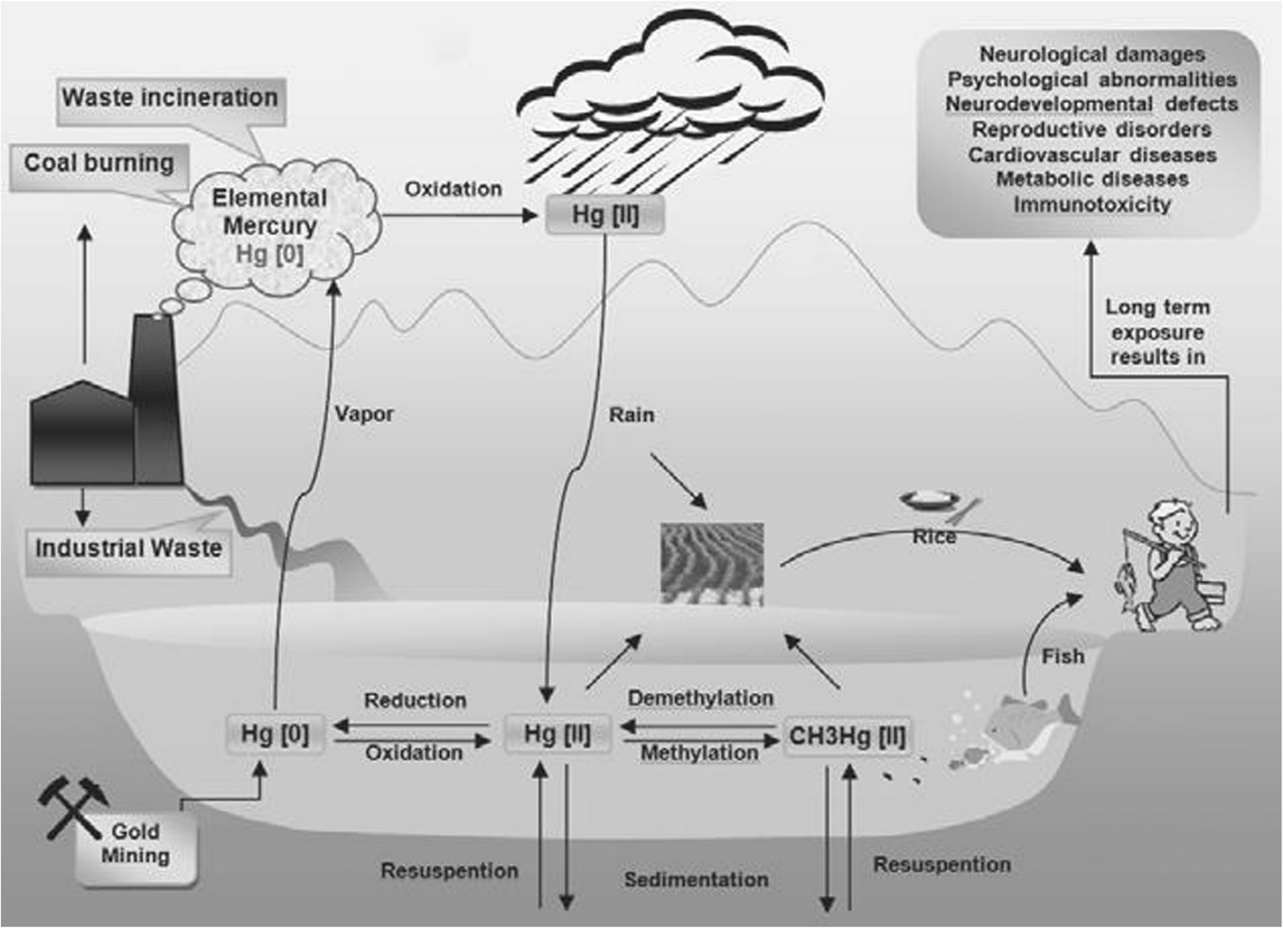
**Schematic view of mercury environmental recycling from the atmospheric emission, deposition, exposure and bioaccumulation, Hg [0]: (elemental mercury), Hg [II]: (inorganic mercury) and CH3Hg [II] (retrieved with permission from: [**[9]**].**

Mercury could be found in different commercial forms [8]. Mercury and its related compounds are being circulated and concentrated in soil and distributed into the air via coal fuels, industrial furnaces or active volcanoes. It then returns to the soil, water, or living organisms. Recycling from atmospheric emission, deposition in water reservoirs and exposure and bioaccumulation in animals and humans is a known example of mercury cycle in the environment [9] (see Figure 1).

### Epidemiology

Humans exposure to mercury usually take place via eating mercury contaminated food, dental care procedures (using amalgams in endodontics) using mercury based, thermometers, and sphygmomanometer), occupational exposure (e.g. mining) and others (using fluorescent light bulbs and batteries) [10].

Metallic mercury intoxication was known in ancient times by Aristotle, but that large scale occupational poisoning with mercury had occurred when the great statue of Buddha of Nara was constructed in 8^th^ century in Japan [11].

In 20^th^ century, two big disasters of mercury poisoning had been reported. The first, Minamata disease; poisoning of 2200 peoples due to consumption of mercury contaminated fishes and shell fish in Kyushu Japan. Also other cases of mercury intoxication had been reported in Niigata (the main island of Honshu, Japan) with approximately 700 victims, during 1950^,^s and 1960^,^s [9,12-14].

The third, three epidemics of mercury poisoning cases have been reported in Iraq during 1955-1956, 1959-1960, and largest outbreak in 1971-1972 in the rural population following the consumption of mercury contaminated homemade. Based on official reports, 6530 patients were hospitalized and 459 persons died [13,14].

There is a serious concern of environmental pollution following handling of mercury compounds, for example; dumping inorganic mercury along the Amazon River in Brazil, pit-working in gold mines in Tanzania, Indonesia, and the Philippines, Ecuador, Faroe islands, French Guiana, New Zealand, Peru, Seychelles island and Slovenia [15,16].

Some sea foods e.g. tuna fish may concentrate mercury compounds and its chronic use may cause poisoning. Similarly, in coastal provinces of Iran (e.g. Khuzestan) [17-19] mercury compounds have been reported in tap and agricultural water sources in Shiraz and Mashhad, two populous cities in Iran [20,21]. Persian Gulf and Caspian Sea are the main seafood sources in Iran. Although, mercury concentration in marginal countries of Caspian Sea except the Republic of Azerbaijan and with coast line of the Persian Gulf and Oman Sea are generally quite low by international standard, but large consumption pattern may result in increased health risks [22-24].

According to recent studies it has been shown that mercury vapors from handling of amalgam, can be hazardous for dental staffs [25,26].

Mercury poisoning is a known topic in various regions of the world, but the incidence of new cases of poisoning in Iran has created an opportunity to reconsider to this silent threat.

Governmental and nongovernmental organizations should prepare basic data about mercury poisoning and design informative and educational programs on mercury poisoning in order to substantially reduce the incidence of poisoning with mercury. This review can be helpful in achieving the purpose to manage the various aspects of poisoning by mercury compounds.

### Mechanisms of toxicity

Mercury compounds exert toxic health effects by different mechanisms such as; interruption of microtubule formation, changing intracellular calcium balance and membrane potential, altering cell membrane integrity, disturbing or inhibition of enzymes, inducing oxidative stress, inhibition of protein and DNA synthesis and disturbing immune functions [27].

Mercury binds to phosphoryl, carboxyl and amide groups in biological molecules [28]. Methyl mercury induces oxidative stress and the free radicals may cause neurotoxicity. On the other hand, it has been reported that accumulation of serotonin, aspartate, and glutamate has a role in mechanism of methyl mercury induced-neurotoxicity [29].

Methyl mercury is converted to inorganic form in CNS that binds to sulfhydryl-containing molecules. Inorganic mercury and methyl mercury bind to thiol-containing protein e.g. glutamine, cysteine, albumin and etc. These complexes affect the distribution of mercury in the body [30].

Binding to endogenous thiol-groups facilitate the distribution of mercury compounds in the body. It also protects the compounds from binding to other proteins, thus providing a protective mechanism [31].

### Clinical manifestations

Different forms of mercury compounds have different clinical manifestations and adverse effects that will be explained in the details below.

### Elemental mercury

Inhaling elemental mercury vapors causes acute symptoms such as cough, chills, fever, and shortness of breath, and also GIT complaints such as nausea, vomiting and diarrhea accompanied by a metallic taste, dysphagia, salivation, weakness, headaches and visual disorders [28]. Long-term inhalation of elemental mercury may cause cognitive impairment including decreased performance intellectual functioning, impairments of attention and short term memory, visual judgment of angles and directions, psychomotor retardation and personality changes including depression and willing to be alone, anxiety and lack of sensitivity to physical stimuli [32]. Whenever elemental or metallic mercury is ingested, it rarely confronts to clinical consequences in normal GIT mucosa. However, people having abnormal GIT mucosa absorb enough elemental mercury during exposure, producing severe irritation [2]. Corrosive injuries will start immediately after mercuric salts ingestion. Oral cavity problems such as inflammation of the mouth, ulcerative gingivitis, loose teeth, gingival bleeding and metallic taste may be observed [33]. Also, a grayish discoloration of mucous membranes, nausea, vomiting, local oropharyngeal pain, bloody diarrhea may be seen. Released mercury from dental amalgams may even induce stomatitis [34,35].

Elemental mercury crosses the alveolar membrane during respiration and readily absorbs into blood, and then distributed and transferred into the tissues [36]. The clinical manifestations of intoxication include: chest pain, dyspnea, dry cough, hypoxemia and altered carbon monoxide diffusing capacity and ventilatory patterns [37]. Mercury vapor at higher concentrations have caused necrotizing bronchitis, bronchiolitis and pneumonitis. It can also progress to pulmonary edema, respiratory failure and death. Complications include multiple pneumothoraces, pneumomediastinum, and subcutaneous emphysema [38]. In survivors, severe pulmonary complication like; interstitial fibrosis and residual restrictive pulmonary diseases may be developed [28]. Subcutaneous injection of a solution containing metallic mercury may cause local abscess and granuloma formations. IV (intravenous) injection cause acute pulmonary embolism and systemic microembolism with respiratory failure [38].

### Inorganic mercury

In acute cases ingestion of inorganic mercury salts cause gastroenteritis. The color of mucous membranes changes rapidly along with development of metallic taste, local oropharyngeal pain, nausea, vomiting, bloody diarrhea, colic abdominal pain and renal dysfunction [15].

Subsequently, stomatitis, hematemesis, and hematochezia may be seen, chronic inorganic mercury salts intoxication may lead to development of tremor of the lips, tongue, severe salivation, losing teeth, anorexia, and weight loss [28,39].

In addition to salivation, gingivitis, gingival bleeding, oral stomatitis, corrosive damage to the mouth and throat have also been observed. The symptoms of acute inorganic mercury inhalation include dyspnea, chest pain, tightness, and dry cough, which are followed by acute chemical pneumonitis and bronchiolitis. Another clinical manifestation is shock that causes to massive fluid loss, and acute tubular necrosis. The predominant manifestations of sub-acute or chronic mercury intoxication include GI symptoms, neurologic abnormalities and renal dysfunction [15,28,39].

The first visible lesion of atherosclerosis in arterial wall is the fatty streak or the foam cell. These cells penetrate into the sub-endothelial space. This penetration can predispose uptake and storage cholesterol, and may result in atherogenesis [40]. Oxidation of LDL in the arterial intima has an important role in athrogenesis. Oxidized LDL increases pro-inflammatory genes expression that causes to aggregate monocyte in the vessels wall and may induce vessel dysfunction. This behaves to generate free radicals [41]. Mercury compounds catalyze peroxidation, e.g. mercuric chloride increases hydrogen peroxide formation and depletes glutathione. This process is increased at the risk of coronary heart disease [42]. The clinical finding of mercury toxicity include coronary heart disease (CHD), myocardial infarction (MI), increases in carotid intimal medial thickness (IMT), carotid obstruction and hypertension [43].

When inorganic mercury compounds are absorbed into bloodstream, the highest concentration (about 85-90%) is found in the kidneys. Inorganic mercury salts are taken up and accumulated in the proximal tubules of the kidneys [44,45]. Clinical findings are polyuria and proteinuria (especially low molecular proteinuria) which are the main indicators of tubular damage in kidneys. In severe conditions, patients suffer from nephrotic syndrome with hematuria and anuria [44]. Chronic inorganic mercury exposure can cause immune complex nephritis, especially membranous nephropathy [46]. In humans, long term exposure to mercury has been accompanied with immunological glomerular diseases which is responsible for mercury-induced nephropathy [47].

### Organic mercury

In mild exposure, organic mercury compounds especially methyl mercury do not produce severe symptoms, but high exposure to organic mercury compounds leads to acute GIT symptoms and delayed neurotoxicity, regional destruction of neurons [28,48]. Ethyl mercury is rapidly metabolized into inorganic mercury that can induce nephrotoxicity [49]. CNS manifestation including autism syndrome which may appear after ethyl mercury intoxication [50,51]. Poisoning with organic mercury often occurs after eating some sea food containing mercury. Organic mercury is divided into three forms; aryl, short chain alkyl, and long chain alkyl compounds. Aryl and long chain alkyl have similar properties to inorganic mercury toxicity, but they are slightly corrosive (organic mercury is less corrosive than inorganic mercury) [33,52]. Also, in contaminated areas, most of the fresh water and salt water fish contain methyl mercury and this agent has acute GI symptoms, in which the above subjects have been pointed out [52].

### Toxic effects on CNS

Toxic effects of mercury compounds on the human central nervous system are well known and in experimental animals it has been shown that mercury cross the placenta and reach fetal brain and get accumulated in the CNs and subsequent neurological disturbance occur in fetus [53,54].

### Toxic effects on skin

Mercury compounds show toxic effects on the skin in many ways. Most common symptoms of contact dermatitis after exposure to mercury compounds include mild swelling, vesiculation, scaling, irritation, urticaria and erythema. Allergic contact dermatitis accompanied by pain, is the most important form of mercurial reaction in skin that can occur by both topical and systemic exposure [34]. In case of injection these mucocutaneous hyperpigmentation results and also purpura may be seen in advanced stage [34,35].

### Diagnostic evaluation

Mercury exists in several physical and chemical statuses and may undergo biotransformation. In clinical laboratories, mercury levels in blood and urine are often determined as the total mercury, without paying attention to the physical and chemical forms [55].

### Urine

Urine is a good sample for assaying elemental and inorganic mercury. Quantity more than 100 μg/L, produce neurological signs while concentration greater than 800 μg/L are often associated with death. Organic mercury such as methyl mercury is excreted mainly in feces, so the urine sample test is not a reliable indicator of the level of organic mercury in the body [56].

### Blood

In acute intoxication, concentration of methyl mercury in red blood cells is high, but it varies in chronic toxicity. The whole blood mercury concentration is often less than 10 μg/L, but it normally may reach to 20 μg/L. After long term exposure to mercury vapors, the blood mercury concentration may be increased to 35 μg/L [27].

### Hair and nail

Hair has high sulfhydryl groups and mercury compounds have high tendency to bind sulfur. After exposure to methyl mercury, total mercury levels in hair and blood will be used as biomarkers to evaluate the extent of poisoning. Hair: blood ratio in human is 250:1 [57]. It may be noted that hair analysis should not be used alone for confirming mercury exposure or its toxicity. In general, mercury concentrations in the hair do not exceed 10 mg/kg. In moderate intoxications, hair mercury concentration is in the range of 200-800 mg/kg but in severe case it may reach to 2400 mg/kg. WHO has advised monitoring of hair levels of methyl mercury in pregnant women and the concentrations equal or greater than 10 ppm increase the risk of neurological deficits in the next generation [33]. Meanwhile, the Methyl mercury easily crosses the blood-placenta barrier and accumulates more in the fetus than the mother. Methyl mercury binds to hemoglobin, its concentration in cord blood is higher than mother’s blood. The umbilical cord is formed and developed mainly in the second and third trimesters. Both inorganic and methyl mercury are detected in the regular analysis of total mercury concentration, but the methylated form of mercury is detected in cord blood. Based on this result, the National Research Council (NRC) recommended the cord blood mercury concentration as the best available biomarker for fetal exposure to methyl mercury [58,59]. According to Brockman et al. [60] report the Hg/Selenium molar ratio is suggested to assess methyl mercury exposures in rats’ nails. Therefore, in human studies the nails may simultaneously be used to evaluate methyl mercury exposures [60].

### Application of nano-medicine in the diagnosis of mercury poisoning

Nanotechnology has revolutionized drug and medical sciences. This technology can cover a wide spread application such as tissue, cell and gene structures, medical instruments and tools, drugs delivery [61] and in biomedical researches, diagnosis evaluations and treatments [62]. Efficient methods have been presented in the literature regarding diagnosis of mercury poisoning. The use of gold nanoparticles (AuNPs) which is a rapid, cheap and sensitive detection method. This method detect DNA and RNA sequences [63,64]. Some studies have reported that Hg^2+^ bind thymine (thymine-mercury-thymine). This interaction may form base pairs in DNA. These bases are absorbed onto AuPNs surfaces. This combination develops Hg2+ sensors, based on the function of DNA and lysozyme gold. After the combinations of DNA with gold nanoparticles, then Hg2+ is detected by colorimetric sensors [63].

Silver nanoparticles (AgNPs) have been used as antibacterial, sunscreens and cosmetic agents. Also, AgNPs have been used as a biosensor or sensitive detector of low concentration Hg2+ ions in homogeneous aqueous solutions [65]. On the other hand, ultrasensitive surface-enhanced Raman scattering (SERS) nanosensor developed for mercury ion (Hg^2+^) detection based on 4-mercaptopyridine (4-MPY) functionalized silver nanoparticles (AgNPs) (4-MPY-AgNPs) in the presence of spermine [66]. This reagent determine mercury up to 0.34 nM. Other Sensors for Mercury (Hg, HgI, HgII) determine concentration levels up to parts-per-billion [67,68].

### Other diagnostic evaluation

Diagnostic tests depend on the clinical situation that includes: complete blood cell count, electrolytes assay, renal and liver function tests initially in acute elemental mercury vapor. Chest X ray (CXR) may show interstitial or alveolar abnormalities. Thereafter, acute respiratory distress syndrome (ARDS) may appear. Other diagnostic procedures are electrocardiography (ECG), pulmonary function test (PFT), cardiovascular monitoring, electroneuromyography and neuropsychologic tests [27].

### Treatment of mercury poisoning

#### Immediate considerations and decontamination

Monitoring of vital organs is needed in primary management of acute exposure to elemental mercury vapors. Supplemental oxygen or endotracheal intubation and mechanical ventilation are recommended [38]. After pulmonary aspiration bronchial lavage must not be done, because the particles of mercury can disperse further into the lungs and the level of absorption may increase. Chest X-rays determine the extent of dispersion. The small mercury droplets are absorbed faster than the bigger ones [39]. In acute ingestions of inorganic mercury, vascular access for IV fluid replacement is required to prevent shock. Inorganic mercury produces severe corrosive injuries. For corrosive injury, endoscopic examination is needed because it causes oropharyngeal edema and upper airway obstruction. To overcome obstruction of the airway, IV fluid therapy and endotracheal intubation and/or tracheostomy are needed [38,39]. The skin should be washed with soap and water in case of direct contact with mercury.

Gastrointestinal decontamination should be implemented for inorganic mercury salt because of systemic absorption, but the important problem is the corrosive property of these compounds. In spite of the corrosiveness of inorganic mercury and the risk for perforation, the removal of inorganic mercury is still beneficial. Whole-bowel irrigation with polyethylene glycol solution may be useful for removing residual mercury. Serial abdominal radiographies are needed to follow -up of the patients [28]. Activated charcoal (AC) may be used but its efficacy is controversial in case of mercury poisoning. The usual oral dose of AC is 0.5-1 gr/kg, with a maximum dose of 100 gr [69]. Many organic and inorganic contaminants are removed with this method [70]. It is believed that AC has been used to absorb different agents, except hydrocarbons, acids-alkalis, ethanol and heavy metal. Unlike in the cases of heavy metal poisoning, charcoal tightly binds with metallic compounds [38,69].

### Chelating agents

#### Penicillamine

It is a white crystalline, water-soluble derivative of penicillin. D-penicillamine (DPA) is preferred to L isomer, because DPA is less toxic than the L isomer [71]. D-penicillamine is the drug of choice in Wilson’s disease, and also useful in the management of other heavy metal toxicity [72].

Penicillamine increase urinary excretion of lead and mercury. The dosage schedule of DPA is: adults 250 mg qid, po, for 1-2 weeks, children 20-30 mg/kg/daily in 4 divided doses (maximum 250 mg/dose). D-penicillamine is only used for elemental and inorganic mercury toxicity and is not useful for organic mercury toxicity [73]. Hypersensitivity and nephrotoxicity are the most common adverse effects of penicillamine [71]. N-acetyl-d, l, penicillamine (NAP) is an analog of DPA, that it is more effective chelator of mercury. Recently succimer has replaced penicillamine, because of its strong metal-mobilizing capacity and lower side-effects [27,71].

### Dimercaprol or British anti-Lewisite (BAL)

**Figure 2:**
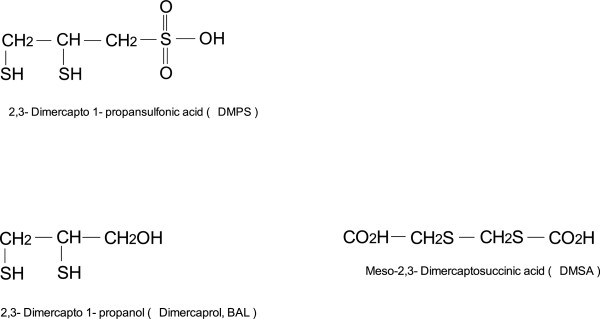
Chemical formula of BAL and their analogs meso-2,3-Dimercaptosuccinic acid (DMSA) and 2,3 Dimercapto-1-propanesulfonic acid (DMPS).

Since more than 60 years, BAL has been prescribed by physicians for the treatment of heavy metal poisonings, both accidental and iatrogenic. During World War II, BAL decreased the risk of damage or death of the allied soldiers. In 1951, BAL was applied to treat Wilson’s disease. Nowadays, BAL is one of the prominent drugs used in the management of heavy metals poisoning [74] (Figure 2).

In poisoning cases with elemental and inorganic mercury salts, dimercaprol (BAL) may be administered 5 mg/kg IM once, 2.5 mg/kg IM every 8 to12 hours for 1 day, and then 2.5 mg/kg IM every 12 to 24 hours for 7 days [38]. Dimercaprol is ineffective and it may even increase mercury levels in the brain and aggravate CNS symptoms in case of organic mercury poisoning [75]. The common side effects of dimercaprol include nausea, vomiting, hypertension, tachycardia, pain at the injection site, headache, diaphoresis and convulsions [38].

### Meso 2,3-dimercaptosuccinic acid (Succimer,DMSA)

It is a water-soluble analog of BAL, with chemical formula C_4_H_6_O_4_S_2,_ approved by FDA in 1991. Meso 2,3-dimercaptosuccinic acid inhibit activity of sulfhydryl-containing enzymes and prevents mercury induced symptoms [71,76]. In humans, *DMSA* is rapidly metabolized and excreted via urine and a small amount via bile and lungs [77]. In the United States, BAL or DMSA is preferred for treatment of inorganic mercury poisoning [28]. WHO recommends that DMSA should be started in children with urine mercury levels equal or greater than 50 μg/mL creatinine, if they are even asymptomatic [78]. DMSA has a half-life of 3.2 h [77].

DMSA is given via oral administration or IV injection. Adult doses are 10 mg/kg tid for the first 5 days, then 10 mg/kg bid for the next 14 days. Children dose is calculated based on the body surface area (BSA), 350 mg/m^2^tid for the first 5 days, then 350 mg/m^2^ bid for the next 14 days. If necessary, it may be repeated, with a 2- week interval between treatments [38]. The dimercapto chelating agents are least toxic. But in some patients, neutropenia has been reported, therefore CBC, renal and hepatic functions should be checked before starting and during the treatment. The side effects of DMSA include GI disorders, skin rashes and flu-like symptoms [77].

### 2,3-dimercapto-1-propane sulfonic acid (Unithiol, DMPS)

It is a water-soluble analog of the dimercaprol with chemical formula C_3_H_7_O_3_S_3_Na,. It has been approved for use in Russia and other former Soviet countries since 1958, in Germany since 1976, and in the USA since 1999 [71,76]. It has replaced DMSA in Europe [28]. In the body, DMPS is oxidized to disulfide forms. At least 80% of DMPS is oxidized within the first 30 minute. Approximately, 84% of total DMPS is excreted by the renal system. It reduces the renal mercury burden when it enters the renal tubular cells [79]. DMPS penetrates into the kidney cells, and removes the mercury accumulated in renal tissues and excrete mercury into the urine. Based on clinical and experimental evidences, it has been shown that DMPS remove mercuric mercury deposits in human tissues except brain [76,77]. All of the complexes of inorganic mercury and chelating agents are excreted via renal system [80].

DMPS is given either orally or IV injection. The adult dose is: 250 mg IV every 4 hours for the first 48 hours is administered, then 250 mg IV every 6 hours for the second 48 hours and next 250 mg IV every 8 hours. After IV administration, oral therapy may be started with 300 mg tid for 7 weeks. Duration of treatment depends on the concentration of mercury in blood and urine. Side effects are rare but, rash, nausea, and leucopenia may be observed [38].

### New DMSA analogues

**Figure 3:**
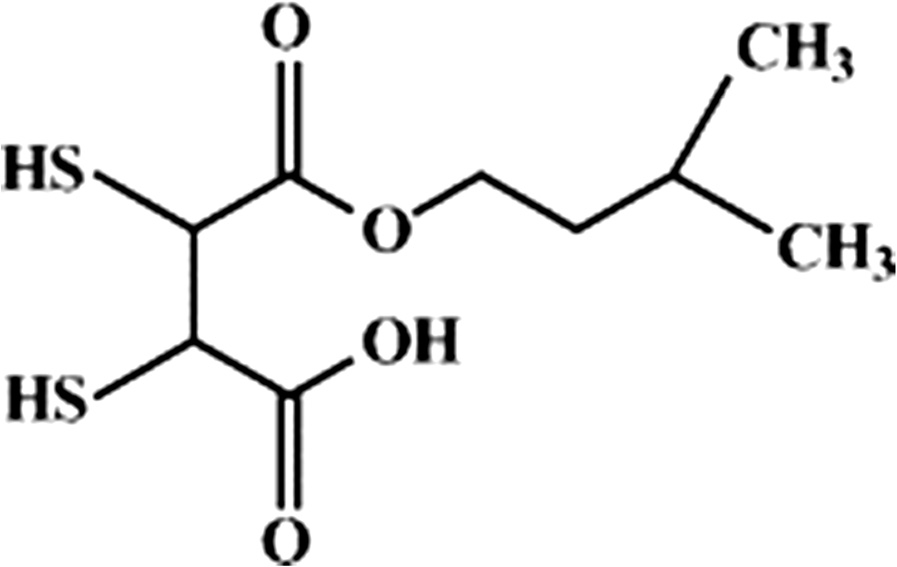
Chemical formula of MiADMSA (mono isoamyl ester ofdimercaptosuccinic acid).

Recently, many studies have shown that esters of DMSA may be more effective antidotes for heavy metal poisoning. These compounds are mono and di esters of DMSA that can enhance tissue elimination of mercury [81]. DMSA removes mercury both from the kidneys and bile. Its sulfhydryl group binds very tightly to mercury [82]. DMSA has hydrophilic and lipophobic properties. It cannot pass through cell membrane. *Mono isoamyl ester of DMSA (MiADMSA)* is a water-soluble lipophilic chelating agent and it is C_5_ branched chain ester (Figure 3). It may be a more effective chelating agent for reducing lead, mercury and cadmium burden [83]. MiADMSA can penetrate to intracellular space and has an extensive cellular distribution. It removes heavy metals from both intra and extra cellular sites [84].

MiADMSA can decrease the oxidative stress in tissue by two ways. First, it removes the heavy metal from the target organ and second, it scavenges ROS (reactive oxygen species) via sulfhydryl groups [84]. Heavy metals such as mercury, lead and selenium have a high affinity for sulfhydryl groups [85]. MiADMSA has lipophilic property and its molecular size may allow removing heavy metals and producing better therapeutic efficacy [83]. It is administrated via oral and intraperitoneal route at doses 25,50 and 100 mg/kg. Although, based on histopathological studies of liver and kidneys in experimental animals, oral administration has been found better than intraperitoneal injection [81].

**Figure 4:**
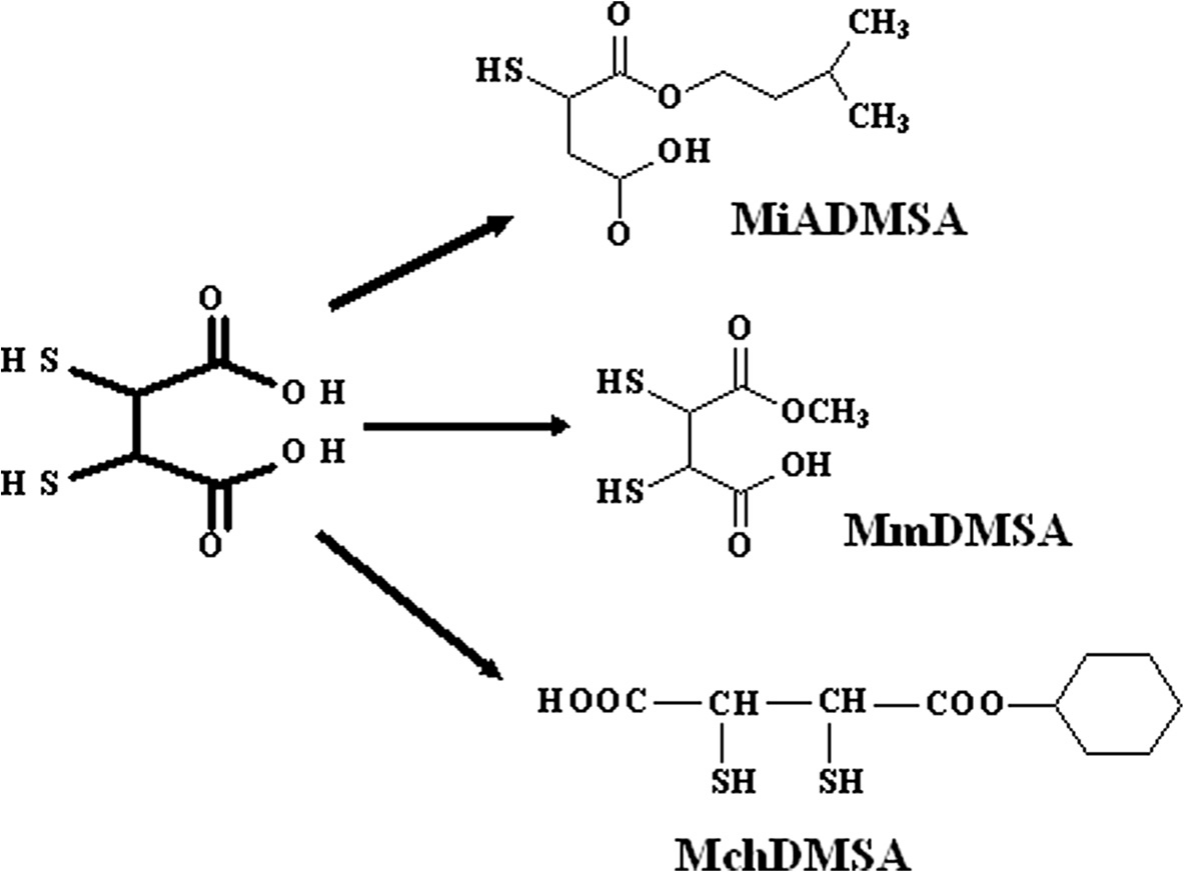
New monoesters of dimercaptosuccinic acid (DMSA).

Other new DMSA analogous are *Monomethyl DMSA (MmDMSA)* and *Monocyclohexyl DMSA (MchDMSA)*. MmDMSA has a straight and branched chain of methyl groups, whereas MchDMSA has a cyclic carbon chain (Figure 4). Both are lipophilic and penetrate into cells. Both of them are chelating agents and are administrated through oral route. However, more studies are required to evaluate their efficacy [86].

### Combination therapy with chelating agents

Nowadays, one of the main subjects in the treatment of heavy metal toxicity is the combination therapy. Co-administration of DMSA with MiADMSA has been found more effective than mono-therapy with MiADMSA, not only in controlling lipid peroxidation but also in controlling decreased catalase activity. It helps to reduce the dose of chelator agent, provides better clinical recoveries and minimize the possible side effects [81,87].

### Plasma exchange-hemodialysis-plasmapheresis

Plasma exchange is initiated about 24-36 hours after the clinical diagnosis, when the patient’s life is in danger and there is no suitable alternative therapy. Plasma exchange may be used in emergency condition, if there is a high plasma concentration of pathogenic substances. Therefore, plasma exchange can potentially be useful in heavy metals toxicity e.g. mercury [88]. Hemodialysis is the best way for water-soluble and dialyzable substances, also if renal failure occurs, hemodialysis maybe necessary [38,89]. Some of the toxic substances can strongly bind to plasma proteins and cannot be removed by hemodialysis. Plasmapheresis is eventually able to remove protein- bound heavy metals in plasma, such as mercury. Some toxicologists suggest using these procedures with chelating agents. In mono-therapy the elimination half- life of inorganic mercury may vary from 30 to100 days. When DMPS and hemodialysis are co-administered, the elimination half-life may be decreased between 2 to 8 days [89,90].

### Managements of mercury contaminations

#### Natural and chemical decontamination

Algae, Azolla and other aquatic plants possess the ability to uptake toxic agents from the environment [91,92]. Chlorella increases elimination of mercury from the GIT, muscles, ligaments, connective tissue, and bone [93]. Chlorella and cilantro as food materials can detoxify some neurotoxins such as heavy metals (e.g. mercury) and toxic chemicals (e.g. phthalates, plasticizers and insecticides [94,95]. Photoinduced electron transfer (PET) sensor has been used for the detection of mercury ions [96]. It shows high selectivity for mercury ions in buffer solution (pH = 7). This sensor can selectively bind to very low concentration Hg^2+^ to form stable complexes [96]. The complexes of AgNPs with polyethylene glycol (PEG) and polyvinylpyrrolidone (PVP) are other systems that show high selectivity for Hg^2+^[65].

### Nano filtration

Nanotechnology may help to decrease water pollution problems by removing microorganisms, pesticides, insecticides and heavy metals (lead, mercury, cadmium, zinc). Nano-catalysts and nano-filters can eliminate toxic contaminants from waste waters [97]. Removing mercury by carbon nanotubes (CNTs) is an effective method in this field. Oxidized CNTs can absorb cations, because in this form the surface of absorption of CNTs is increased along with chemical and electrostatic binding [98].

Finally, long term administration of nano-medicines is still waiting to be approved by FDA and other authorized international organizations. In addition, removal of vapor-phase elemental mercury from stack emissions with sulfur-impregnated activated carbon has been under consideration.

## Conclusion

Mercury exposure leads to harmful effects on almost every organ and system. It should be considered as a silent threat to environment and human life, through the world. The main concern is with the more subtle effects arising from prenatal to adult's period, and exist delay development and cognitive changes in children and clinical manifestations in adults. New protocols for the treatment of poisoning such as access to new antidotes, chelating agents, combination therapy of different chelating agents and specific nano-sorbents can help in the management of mercury poisoning. There are risks of mercury compounds for health in the worldwide. Therefore governmental and non-governmental organizations need to identify highly prone people to mercury exposure, and make sure safe food and drinking water.

In addition, it is necessary to pay attention to the safe transport and handling of mercury compounds.

## Competing interest

The authors declared that they have no competing interests.

## Author contributions

MRR: drafted the review article; MRR: searched in literature and arranged the information; SK: arranged references and edited figures and inserted the references using endnote software; AAM; he is corresponding person and carried out the final writing and editing of the manuscript, sending the manuscript, he responded to the reviewers and corrected and supervised all parts of the manuscript writing All authors read and approved the final manuscript.
